# Systematic Interrogation of the Temperature Perturbation in the Insulin Signaling Pathway for Optogenetic Stimulation

**DOI:** 10.3390/cells11193136

**Published:** 2022-10-05

**Authors:** Qi Dong, Mizuki Endo, Genki Kawamura, Takeaki Ozawa

**Affiliations:** Department of Chemistry, School of Science, The University of Tokyo, 7-3-1 Hongo, Bunkyo-ku, Tokyo 113-0033, Japan

**Keywords:** insulin signaling, NIR, optogenetics, temperature

## Abstract

The application of NIR to optogenetic systems is in great demand due to its superior properties enabling in vivo deep tissue penetration. Irradiation of NIR to tissue samples or cells rapidly generates heat locally. The resultant elevation in temperature affects cells at the molecular level because of the activation of the heat shock pathway and ROS generation. Nevertheless, few reports have presented detailed comparisons of the effects of the temperature change rate on signaling pathway biomolecules, especially those of rapid heat changes. Aiming at broadening the understanding of temperature sensitivity, we investigated seven insulin signaling pathway biomolecules (INSR, IRS1, Akt, GSK3β, p70S6K, FoxO1, and ERK1/2) in three cell lines (C2C12, HepG2, and Fao) at temperatures between 25 and 45 °C. The results show that, except for INSR, pAkt(T308), and FoxO1, biomolecules are sensitive to rapid temperature changes at temperatures higher than 42 °C, at which they are significantly phosphorylated. At 25 °C, around a 50% reduction in phosphorylation occurred. Moreover, p70S6K is sensitive over time. It dephosphorylates quickly (5 min) and then phosphorylates over time. Our findings extend the temperature range to 45 °C, while providing additional time course information about the signaling pathway biomolecule response necessary to advance NIR optogenetic research.

## 1. Introduction

Genetically engineered protein research has received vast attention, flourishing and advancing in recent decades. Optogenetic approaches, including those using fluorescent proteins (FPs), biosensors, optogenetic tools, and reporters, offer high spatiotemporal accuracy. They have inspired numerous innovative ideas to expand our understanding of organism functions. Nevertheless, the conventional usage of blue light hinders its application to living animals because of light scattering and absorption by hemoglobin, water, and melanin inside the tissues. In contrast, at the near-infrared (NIR) window (NIR-I of 600–900 nm and NIR-II of 900–1800 nm), the absorption of light by hemoglobin and water is minimal [1,2]. Therefore, to advance the studies noninvasively into the deep tissue, recent research has assessed various methods of using NIR with the ability to maximize the in vivo penetration depths [3,4,5].

Bacterial phytochromes (BphPs) with heme catabolic intermediate biliverdin (BV) as their chromophore are widely adopted NIR FPs that have developed into dimer systems with variations such as iRFP670, iRFP713, and iRFP720 [6,7,8]. Other NIR FPs include allophycocyanins (APCs) such as smURFP and iRFP670, as well as cyanobacteriochrome (CBCR)-based miRFP670nano that are available in a smaller size [9,10,11]. Based on their properties, NIR-based biosensors and optogenetic tools have also been engineered, such as NIR-GECO1 to sense the Ca^2+^ channel, the development of APC-modified nanoCRISPR to activate CRISPR-Cas9 optogenetically, and RpBph-P1-based protein targeting control and transcription activation in cells [12,13,14,15,16,17]. Moreover, NIR can be used in combination with upconversion nanoparticles to convert NIR to blue light to use existing optogenetic tools [4,18,19,20].

The irradiation of NIR to tissue or cell samples of interest rapidly generates heat locally through photothermal interaction. Head tissue modeling studies have predicted local temperature increases of up to 10 °C, depending on laser spot sizes and intensities [21]. Despite channeling the generated heat favorably for cancer treatment, the heat generated by NIR affects tissue functions. At the cellular level, the temperature increase is a stressor that subsequently induces heat shock protein activity at temperatures higher than 42 °C [22,23,24,25,26]. The main subgroup protein of the family that regulates the MAPK-ERK pathway, 90-kDa heat-shock protein (Hsp90), interacts with Akt kinase (Akt) and glycogen synthase kinase 3 (GSK3) to influence cellular activities [27,28,29,30,31]. Heat stress also disturbs the mitochondrial antioxidant system, leading to intracellular ROS production and cell apoptosis [32,33]. Earlier studies have shown that rapidly increased local temperatures alter plasma membranes’ electrical capacitance, which consequently affects cellular metabolism [34]. The absorption of NIR by water generates heat. However, details of the influences of the rate of temperature change on the phenomenon described above have rarely been studied directly, and have never been reported. The corresponding responses of surrounding biomolecules must be investigated further.

Signaling pathways are one of the important optogenetic applications. They offer great potential for applying NIR to enhance in vivo penetration depths into tissues of living animals. Some earlier studies have examined the rapid temperature changes caused by NIR and their effects on biomolecules in specific signaling pathways. Recent research often lacks sufficient information about experimental temperature conditions. Elucidating the temperature sensitivity of biomolecules is, therefore, important (1) to eliminate temperature-related factors that lead obtained results away from the truth, (2) to increase awareness of temperature sensitivity to facilitate evidence-based experiment design for additional studies, and (3) to inspire future research of detailed mechanisms in this area.

As described herein, aiming at broadening the understanding of temperature sensitivity of signaling pathway biomolecules, and taking the insulin signaling pathway as an example, we investigated temperature change rates, especially rapid temperature changes and their effects on key biomolecules. Three cell lines that originate from different mammalians (mouse myoblast cell line (C2C12), human liver cancer cell line (HepG2), and rat hepatoma cell line (Fao)) that are used widely for insulin research were selected to examine the phosphorylation changes of key biomolecules including Insulin receptor (INSR), Insulin receptor substrate 1 (IRS1), Extracellular signal-regulated kinase 1/2 (ERK1/2), Akt phosphorylated at S473 (pAkt(S473)), Akt phosphorylated at T308 (pAkt(T308)), p70 Ribosomal S6 kinase (p70S6K), GSK3β, and Forkhead box protein O1 (FoxO1). Cells that underwent rapid stimulation within 5 min to reach the target temperature (25–45 °C) were collected. They showed a significant increase in IRS1, pAkt(S473), GSK3β, and ERK1/2 phosphorylation from 42 °C, whereas dephosphorylation occurs at 25 °C. In all cell lines, p70S6K behaviors were both temperature-sensitive and time-sensitive where a decreasing trend with a rapid temperature increase was observed. By contrast, INSR, pAkt(T308), and FoxO1 were not sensitive to temperature change. This study examined the biomolecule-specific temperature sensitivity in the insulin signaling pathway.

## 2. Materials and Methods

### 2.1. Reagents

#### 2.1.1. Chemical Reagents

The chemical reagents used in this paper include: Tris-(hydroxymethyl)-aminomethane and hydrochloric acid buffer solution (Tris HCl), sodium chloride (NaCl), sodium dodecyl sulfate (SDS), ethylenediaminetetraacetic acid (EDTA), glycerol, 2-mercaptoethanol, hydrochloric acid (HCl), methanol, sodium deoxycholate, bromophenol blue, sucrose, ammonium peroxodisulfate, glycine, Tween^®^20, albumin from bovine serum globulin (BSA), paraformaldehyde (PFA), and insulin (Wako Pure Chemical Industries, Ltd., Osaka, Japan). Gelatin from cold-water fish (Sigma-Aldrich, St Louis, MO, USA) and Triton X-100 (Nacalai Tesque Inc., Kyoto, Japan).

#### 2.1.2. Antibodies for Western Blot and Immunofluorescence

Specific antibodies for pAkt(Ser473)(#4060, 1:5000), pAkt(Thr308)(#4056, 1:2500), Akt(#4691, 1:5000), pINSR(#3024, 1:5000), INSRβ(#3025, 1:5000), p-p70-S6K(#97596, 1:2000), S6K(#9202, 1:5000), pFoxO1(S256)(#9461, 1:2000), FoxO1(#2880, 1:5000), pGSK3β(#5558, 1:5000), GSK3α/β(#5676, 1:5000), p-ERK1/2(T202/Y204)(#9101, 1:5000), ERK1/2(#4695, 1:5000), and Hsp70(#4872, 1:2000) were obtained from (Cell Signaling Technology Inc., Danvers, MA, USA). pIRS1(#09432, 1:2500) and IRS1(#06248, 1:2500) were acquired from (Merck KGaA, Darmstadt, Germany). β-Actin(#A228, 1:5000) was procured from (Sigma-Aldrich). Peroxidase-conjugated secondary antibodies Anti-Rabbit IgG (#NA934, 1:5000) and Anti-Mouse IgG (#NA931, 1:5000) were purchased from (GE Healthcare BioSciences AB, Chicago, IL, USA). Anti-Rabbit IgG Secondary Antibody (Alexa Fluor™ 568, 1:5000) was obtained from (Thermo Fisher Scientific, Waltham, MA, USA).

### 2.2. Cell Culture

Mouse myoblast cell line (C2C12) (CRL-1772; American Type Culture Collection (ATCC), Manassas, VA, USA) was maintained in Dulbecco’s modified Eagle’s medium (DMEM; Nacalai Tesque Inc.) supplemented with 10% fetal bovine serum (Sigma-Aldrich), 100 unit/mL of penicillin (Gibco™, Thermo Fisher Scientific, Waltham, MA, USA), and 2 mM glutamine (GlutaMAX; Gibco™). Human liver cancer cell line (HepG2) (HB-0865; ATCC) cells were maintained in Eagle’s minimal essential medium (MEM; Nacalai Tesque Inc.) supplemented with 10% fetal bovine serum (Sigma-Aldrich), 100 unit/mL of penicillin (Gibco™), and 1% sodium pyruvate (Nacalai Tesque Inc.). Rat hepatoma cell line (Fao) (gifted from Kuroda Lab, SOS, UTokyo) was maintained in Kaighn′s modified Ham′s F12 (Sigma-Aldrich) supplemented with 10% fetal bovine serum (Sigma-Aldrich), 100 unit/mL of penicillin (Gibco™), 2 mM glutamine (GlutaMAX; Gibco™), and 45 mg/L of ascorbic acid (Sigma-Aldrich). After seeding onto a 10 cm dish (Corning Inc., Corning, NY, USA), cells were maintained at 37 °C with 5% CO_2_.

### 2.3. Western Blotting

Cells were seeded on a 35 mm dish (Corning) before the experiment. At approximately 50–60% confluency, they were washed with PBS twice and were replaced with a cell culture medium containing 0.1% FBS for serum starvation for 24 h. Cells were then stimulated accordingly using a tabletop incubator (PIC-101S; AS One Corp.,Osaka, Japan). The incubator temperature was adjusted from 25 °C to 60 °C to allow the cells in different stimulation conditions to reach the targeted temperature at the indicated temperature within 5 min (cells were collected immediately when reaching the target temperature within 5 min). For the constant-temperature experiment, cells were maintained at 25 °C and 45 °C for different timepoints up to 60 min. The temperature changes during the experiment were measured using a thin-fiber temperature sensor (SFS-E-100-ASP) positioned at the bottom of the dish closest to the cells. The sensor was connected with a digital thermometer (model HDS-120E; Anritsu Meter Co., Ltd., Tokyo, Japan). Rapid temperature changes were measured every 1 min while gradual temperature changes were measured at the sampling timepoint.

After stimulation, cells were placed immediately on a metal block positioned on ice and were washed with ice-cold PBS twice. Equal volumes of RIPA buffer (50 mM Tris HCl (pH 7.4), 150 mM NaCl, 1.0% (*v*/*v*) NP-40, 0.5% (*w*/*v*) sodium deoxycholate, 1.0 mM EDTA, 0.1% (*w*/*v*) SDS, and 0.01% (*w*/*v*) sodium azide) with PhosSTOP™ (Roche, Basel, Switzerland) and cOmplete™ (Roche) were added along with 2× sampling buffer (5% SDS, 10% glycerol, 10% 2-mercaptoethanol, and 125 mM Tris-HCl, pH 6.8) immediately to cells for cell lysis. After the collected samples were sonicated (Bioruptor; Cosmo Bio Co. Ltd., Tokyo, Japan) for 3 min, they were boiled at 95 °C for 5 min. The BCA protein assay (Pierce™; Thermo Fisher Scientific) was used to ascertain the protein concentration.

An equal quantity of proteins (8 μg/lane) was loaded to SDS-polyacrylamide gel (12% or 7.5% TGX™ FastCast™, Tokyo, Japan) for electrophoresis. Ponceau (MP biomedicals, Irvine, CA, USA) staining was performed on each membrane to ensure equal protein loading in addition to housekeeping protein (β-Actin). The gels were transferred onto a nitrocellulose membrane and blocked with 2% ECL prime blocking reagent (Amersham; Cytiva, Danaher Corporation, Washington, DC, USA) or 5% Skim milk (Difco™; Becton, Dickinson and Co., Franklin Lakes, NJ, USA) in Tris-buffered saline containing Tween-20 (TBS-T: 150 mM NaCl, 0.05% Tween-20, and 50 mM Tris-HCl, pH 7.6) for 1 h at room temperature. Subsequently, the membranes were blotted with corresponding antibodies at 4 °C overnight. Then, membranes were incubated with corresponding peroxidase-conjugated secondary antibodies for 1 h at RT. Membranes were then developed using ECL^™^ Select Western Blotting Detection Reagent (Amersham; Cytiva, Marlborough, MA, USA) to detect bands with an imager (ImageQuant LAS4000 mini CCD; GE Healthcare) and software (ImageQuant™ LAS 4000mini Ver. 1.2; GE Healthcare).

### 2.4. Immunofluorescence

Cells seeded on a 24-well glass bottom plate were subjected to the same stimulation conditions as those of the Western blot sample. The collected cells were set immediately on a metal block positioned on ice and were washed five times with ice-cold PBS. Then, 4% PFA was added to cells to fix them at room temperature (RT) for 20 min. The cells were then washed with PBS three times and were incubated with 0.2% Triton X-100 in PBS(+) for 15 min at RT. After cells were washed with PBS(+) supplemented with 0.1% Tween-20 (PBST), they were blocked with gelatin from cold-water fish for 1 h at RT. Then, cells were washed twice with PBST and were stained with Hoechst 33342 (1:1000, #62249; Thermo Fisher Scientific Inc., Waltham, MA, USA) for 10 min at RT. After rabbit anti-Akt antibody was dissolved in 2% BSA (in PBST) with 1:200 dilution, it was incubated at RT for 1 h. After washing with PBST another three times, secondary antibodies dissolved in 2% BSA (in PBST) were added to cells for shaking at RT for 1 h. After the final wash with PBST three times (5 min interval), cells were put to immediate microscopic observation using a confocal microscope (FV1200; Olympus Optical Co. Ltd., Tokyo, Japan) with a 10× or 60× lens and imaging acquisition condition: 10 μs pixel^−1^, 568 nm laser excitation, and 405 nm laser for Hoechst 33342, excitation.

### 2.5. Statistical Analysis

The blot image was analyzed using ImageJ ver. 2.3.0 (NIH, Bethesda, MD, USA) and ImageQuant TL 1D Version 8.1 (Cytiva). Data were normalized using antibodies against housekeeping protein (β-Actin). GraphPad Prism software ver. 9.0 was used to perform statistical analysis to report the mean and 95% confidence interval (CI) for each testing condition. One-way analysis of variance (ANOVA) followed by Dunnett’s test was conducted using temperature or time as the variance to compare the difference between test groups.

### 2.6. Image Processing for Article Figures

All the images in this article were processed (representation Western blot images and immunofluorescence) using ImageJ ver. 2.3.0.

## 3. Results

**Figure 1:**
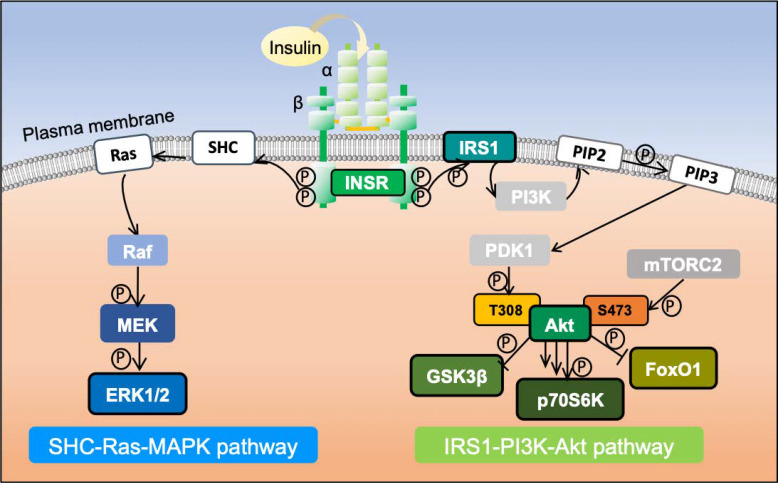
Schematic diagram of insulin signaling pathway activation. Insulin secreted by pancreatic islet beta cells binds to INSR and activates it. The activated cytoplasmic kinase domain of INSR phosphorylates IRS1, triggering different downstream signaling pathways. Among them are the IRS1-phosphatidyl inositol 3-kinase (PI3K)-Akt pathway, which regulates insulin metabolism, and the SHC-Ras-MAPK pathway responsible for growth and differentiation, which are two of the most critical kinase-dependent pathways triggered by insulin stimulation.

The insulin signaling pathway (Figure 1), which functions in regulating blood glucose levels, is a major signaling pathway that is studied extensively. It was, therefore, selected for examination in this study. To explore effect of the rate of temperature change on insulin signaling pathways, seven biomolecules of interest (INSR, IRS1, Akt, GSK3β, p70S6K, FoxO1, and ERK1/2) were investigated in three cell lines (C2C12, HepG2, and Fao cells) that had been starved for 24 h. Two temperature change rates were investigated. For the gradual temperature change, cells were incubated at a constant temperature. In comparison, the rapid rate of temperate change was achieved by positioning cells in an incubator with a greater temperature difference to reach the target temperature rapidly (within 5 min).

### 3.1. Rapid Temperature Increase Elevates the Phosphorylation of Insulin Signaling Pathway Biomolecules, Except for p70S6K

**Figure 2:**
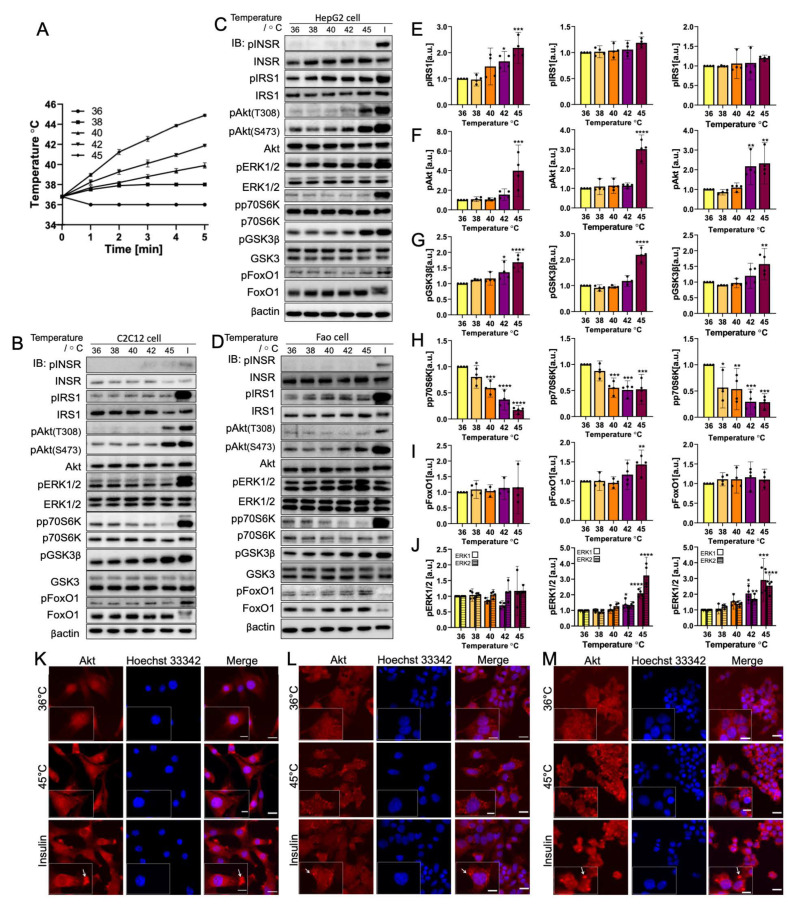
Rapid temperature change rate increases the phosphorylation of insulin signaling pathway biomolecules (IRS1, Akt(S473), GSK3β, and ERK1/2) with the exception of decreases in p70S6K phosphorylation. INSR, pAkt(T308), and FoxO1 phosphorylation are not temperature-sensitive. (**A**) Temperature profile illustration of the cells incubated at 36–45 °C (*N *= 3) within 5 min. Representative immunoblotting image of (**B**) C2C12 cells, (**C**) HepG2 cells, and (**D**) Fao cells. Cells were starved for 24 h and were heated from 36 *°*C to 45 *°*C rapidly within 5 min. Insulin stimulation: 50 nM, 10 min. The phosphorylation change (Left, C2C12 cells; Middle, HepG2 cells; Right, Fao cells) was quantified on (**E**) IRS1, (**F**) pAkt(S473), (**G**) GSK3β, (H) p70S6K, (**I**) FoxO1, and (**J**) ERK1/2. Columns are mean ± 95% confidence interval (*N *= 4 independent experiment). Results were normalized by β-actin (* *p *= 0.0021; ** *p *= 0.0002; *** *p* < 0.001; One-way ANOVA; Dunnett’s test comparing to 36 *°*C). Immunofluorescence analysis of Akt translocation for (K) C2C12 cells, (L) HepG2 cells, and (M) Fao cells. Cells were heated to 45 *°*C (in 5 min) (red) with the nucleus stained with Hoechst 33,342 (blue). Negative control cells were kept at 36 *°*C. Scale bars show 20 μm and 10 μm (zoomed image).

The cells were treated to mimic the rapid heating caused by NIR (Figure 2). The temperature profile within 5 min was measured and is plotted in Figure 2A. In all three cell lines, INSR showed no visible phosphorylation change along with the increase in temperature, which implies a lack of temperature sensitivity in the tested range. In the IRS1-PI3K-Akt pathway, IRS1 and pAkt(S473), together with downstream molecule GSK3β, demonstrated an increase in phosphorylation from 36 °C to 42 °C: the change was significant at 45 °C. pAkt(S473) phosphorylation was elevated up to three-fold (*p* = 0.0002 for C2C12, *p* < 0.0001 for HepG2, and *p* = 0.0021 for Fao). In addition, GSK3β exhibited a two-fold increase (*p* < 0.0001 for C2C12 and HepG2, and *p* = 0.0021 for Fao) for all three cell lines. The results show that IRS1 phosphorylation was significant, with less magnitude for C2C12 (*p* = 0.0002) and HepG2 (*p* = 0.0032) cells. For Fao cells, IRS1 phosphorylate showed a higher mean value at 45 °C than at 36 °C, but the difference was not statistically significant. In addition, pAkt(T308) failed to detect visible changes until 42 °C where a more noticeable increase in phosphorylation was observed at 45 °C. Conversely, p70S6K phosphorylation decreased significantly from 38 °C (*p* < 0.0001) with the rising temperature. However, FoxO1, which Akt also mediates, was not sensitive to the rapid temperature change, showing no statistically significant phosphorylation change. An exception was HepG2, which detected a 1.5-fold increase in FoxO1 phosphorylation (*p* = 0.0002). It is noteworthy that ERK1/2, which belongs to the SHC-Ras-MAPK pathway, was calculated to have a three-fold increase at 45 °C for both ERK 1 and ERK 2 (*p* < 0.0001 at 45 °C), but ERK1/2 in C2C12 cells were not sensitive to the temperature change. Furthermore, Hsp70 was also detected to examine the activation of the heat-shock response pathway by the temperature change (Supplementary Figure S1).

To investigate the activation of the IRS1-PI3K-Akt pathway further and explore the discrepancy in the downstream biomolecule phosphorylation trend, Akt translocation was visualized using immunofluorescence (Figure 2K–M). Insulin-stimulated cells for all cell lines exhibited a concentrated spot-like structure at the cell membrane, indicating the activation of Akt through the IRS1-PI3K-Akt pathway in which Akt translocation occurred. Cells heated rapidly to 45 °C had different Akt translocation patterns. In C2C12 cells, Akt was distributed throughout the cells. By contrast, both HepG2 and Fao cells showed aggregate-like structures that were less intensive than those of the insulin-activated cells. The observation potentially implies noncanonical activation of Akt, independent of the PI3K pathway and of the influence of other signaling pathways.

### 3.2. Gradual Temperature Increase to 45 °C Yielding a Greater Magnitude in the Phosphorylation Increase

**Figure 3:**
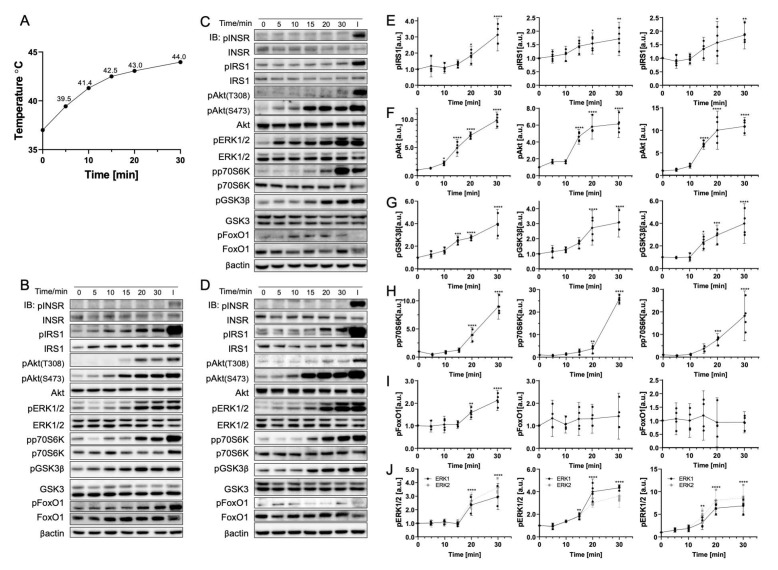
Gradual temperature increase to 45 °C significantly phosphorylates insulin signaling pathway biomolecules. (**A**) Real-time temperature measurement of the cells incubated at 45 °C (*N* = 4). Representative immunoblotting image of (**B**) C2C12 cells, (**C**) HepG2 cells, and (**D**) Fao cells. Cells were starved for 24 h and were positioned in 45 °C incubation from 0 to 30 min. Insulin stimulation: 50 nM, 10 min. The phosphorylation change (Left, C2C12 cells; Middle, HepG2 cells; Right, Fao cells) was quantified for (**E**) IRS1, (**F**) pAkt(S473), (**G**) GSK3β, (**H**) p70S6K, (**I**) FoxO1, and (**J**) ERK1/2. Columns are mean ± 95% confidence intervals (*N* = 4 independent experiment). Results were normalized by β-actin (* *p* = 0.0021; ** *p* = 0.0002; *** *p* < 0.001; One-way ANOVA; Dunnett’s test compared to negative control).

To further understand the effect of the temperature change rate on phosphorylation with a gradual changing rate, cells were positioned in a 45 °C incubator from 5 min to 30 min. The real-time temperature measurement (Figure 3A) recorded the gradual temperature increase from 37 °C to 44 °C during 30 min. This condition corresponds to continuous exposure under NIR light conditions with a continuous increase in temperature. All biomolecules (except FoxO1 in HepG2 and Fao cells) were phosphorylated significantly after 10 min of incubation time. Their temperature was approximately 42 °C. Specifically, IRS1 phosphorylation doubled, and pAkt(S473) phosphorylation increased to around ten-fold (*p* < 0.0001), whereas both GSK3β and ERK1/2 were around four times more phosphorylated (*p* < 0.0001). Similarly to the result reported in Section 3.1, INSR and pAkt(T308) were found to be temperature-insensitive. In addition, pAkt(T308) was only phosphorylated from 20 min onward when the temperature was 43 °C. Contrary to the rapid heating result indicating that p70S6K was dephosphorylated considerably, gradual heating induced a similar reduction in phosphorylation to 50% at 5 min, followed by a notably large increase from 20 min (ranging from 10-fold to 30-fold depending on the cell line). Furthermore, the cell reached approximately 45 °C at 30 min. Compared to the results reported in Section 3.1, the magnitude of changes was generally greater than rapid heating to 42 °C and 45 °C. Both pAkt(S473) and ERK1/2 reached a plateau of the phosphorylation increasing trend at around 15 min.

### 3.3. Rapid Cooling on Cells Causes Minimal Impact on Biomolecule Phosphorylation above 25 °C

**Figure 4:**
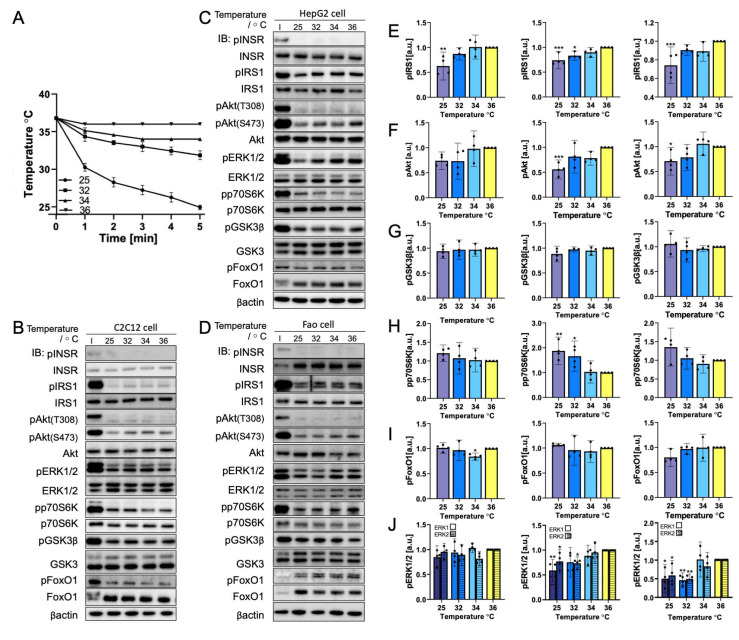
Rapid cooling dephosphorylates insulin signaling pathway biomolecules, except for p70S6K. (**A**) Temperature profile illustration of the cells incubated at 25-36 °C (*N* = 3) within 5 min. Representative immunoblotting image of (**B**) C2C12 cells, (**C**) HepG2 cells, and (D) Fao cells. Cells were starved for 24 h and were cooled rapidly from 36 °C to 25 °C, within 5 min. Insulin stimulation: 50 nM, 10 min. The phosphorylation change (Left, C2C12 cells; Middle, HepG2 cells; Right, Fao cells) was quantified on (**E**) IRS1, (**F**) pAkt(S473), (**G**) GSK3β, (**H**) p70S6K, (**I**) FoxO1, and (**J**) ERK1/2. Columns show the mean ± 95% confidence interval (*N* = 4 independent experiment). Results were normalized by β-actin (* *p* = 0.0021; ** *p* = 0.0002; *** *p* < 0.001; One-way ANOVA; Dunnett’s test compared to 36 °C).

The environment temperature often differs from the NIR heating condition in the testing sample. The cooling event occurs immediately after heating. As a result, rapid cooling was investigated independently in this study. Cells underwent a rapid temperature decrease from 36 °C to 25 °C within 5 min (Figure 4A). The representative Western blot results are displayed in Figure 4. No visible change in phosphorylation was observed from 36 °C to 32 °C for any biomolecule tested in the three cell lines chosen. At 25 °C, IRS1, pAkt(S473), and ERK1/2 were slightly more dephosphorylated than at other temperature points, but INSR, GSK3β, and FoxO1 phosphorylation levels were maintained. By contrast, p70S6K showed an increase in phosphorylation with the decrease in temperature. This observation follows the trend described in Section 3.1 by which p70S6K phosphorylation was found to be temperature-dependent with a rapid temperature changing rate. Quantification results (Figure 4D–I) calculated the average elevation in phosphorylation in p70S6K as up to two-fold. Aside from the increase in mean phosphorylation, p70S6K phosphorylation was only significant in HepG2 cells (*p* = 0.0002). This finding is related to the higher standard deviation of results. Nevertheless, the 95% confidence interval range at 25 °C remained higher than 36 °C, demonstrating the higher phosphorylation level at 25 °C. Furthermore, ERK1/2 (*p* = 0.0021 for HepG2 and *p* = 0.0032 for Fao) dephosphorylation with cooling was significant, except for C2C12 cells. Altogether, ERK1/2 was found to have no significant temperature-dependent phosphorylation change from 25 °C to 45 °C in C2C12 cells.

### 3.4. Gradual Cooling to 25 °C Leads to a Continuous Decrease in Phosphorylation

**Figure 5:**
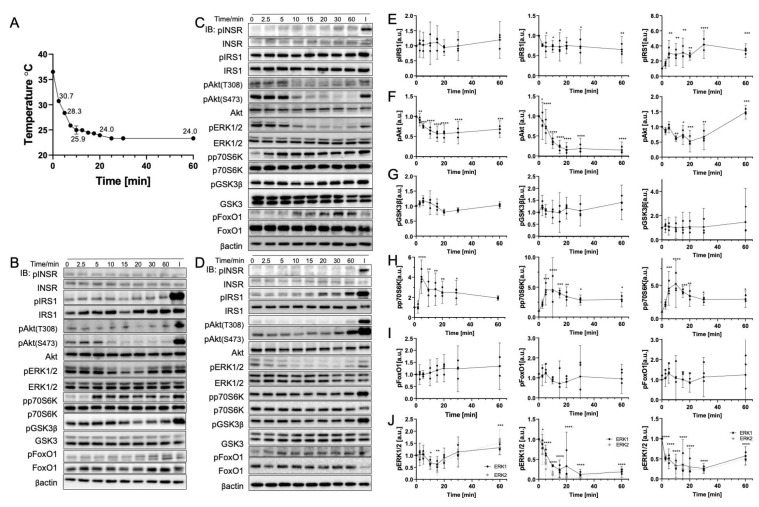
Gradual cooling to 25 °C significantly dephosphorylates Akt and ERK1/2 and phosphorylates p70S6K. (**A**) Real-time temperature measurement of the cells incubated at 25 °C (*N* = 6). Representative immunoblotting image of (**B**) C2C12 cells, (**C**) HepG2 cells, and (**D**) Fao cells. Cells were starved for 24 h and were incubated at 25 °C for up to 60 min. Insulin stimulation: 50 nM, 10 min. Quantification of the phosphorylation change (Left, C2C12 cells; Middle, HepG2 cells; Right, Fao cells) was performed on (**E**) IRS1, (**F**) pAkt(S473), (**G**) GSK3β, (**H**) p70S6K, (**I**) FoxO1, and (**J**) ERK1/2. Columns are mean ± 95% confidence interval (N = 3 independent experiment). Results were normalized by β-actin (* *p* = 0.0021; ** *p* = 0.0002; *** *p* < 0.001; One-way ANOVA; Dunnett’s test compared to negative control).

The rate of temperature change affects biomolecule behavior. Cells were positioned at 25 °C for up to 60 min and were collected at different times to expand our knowledge of the gradual-cooling-related phosphorylation change. In Figure 5A, the temperature measured during the experiment indicates a rapid drop in cell culture medium temperature from 37 °C to 30 °C within 2.5 min. This initial decrease was also reflected in the phosphorylation response as early as 2.5 min. Figure 5E–G illustrate that IRS1, pAkt(S473), and GSK3β from the IRS1-PI3K-Akt pathway were dephosphorylated rapidly. In addition, the loss of dephosphorylation in Akt was more significant than those of other biomolecules. pAkt(S473) in HepG2 was reduced by 70% (*p* < 0.0001), and C2C12 (*p* < 0.0001) and Fao (*p* = 0.0002), respectively, showed 50% and 80% reductions. At 10 min, the temperature reached approximately 25 °C and stabilized for up to 60 min. Compared to the results in Section 3.3, the magnitude of the dephosphorylation at 25 °C in gradual cooling was greater, which was consistent with the phenomenon observed in the heating event. Moreover, p70S6K displayed a notably fourfold increase in phosphorylation at 5–10 min (*p* < 0.0001) and quickly declined to around threefold after 10 min (*p* = 0.0032). FoxO1 failed to display a trend in phosphorylation with time. The data deviation was greater than other biomolecules. In contrast, ERK1/2 were dephosphorylated in HepG2 (*p* < 0.0001 with 70%–90% reduction) and Fao (*p* < 0.0001 with 70% reduction) cells, but less dephosphorylated in C2C12 cells (*p* = 0.0021 with 50% reduction).

## 4. Discussion

This study investigated key biomolecules from insulin signaling pathways to evaluate their sensitivity to different rates of temperature change. The results demonstrated that, at temperatures higher than 42 °C, Akt and MAPK signaling molecules are significantly phosphorylated, but at 25 °C, they are dephosphorylated to around 50%. In addition, INSR, pAkt(T308), and FoxO1 are otherwise not sensitive to temperature fluctuation. Both the gradual and rapid temperature changes induce similar trends of phosphorylation change for biomolecules that are sensitive to temperature change. However, the magnitudes of the changes differ where the gradual temperature change engenders a greater phosphorylation change. For instance, in HepG2 cells, IRS1 dephosphorylation is significant in the condition in which the temperature is decreased gradually to 25 °C in 10 min. A rapid reduction to 25 °C within 5 min shows a reduction in the phosphorylation level, but it is not significant. Similarly, in the heating event, pAkt(S473) phosphorylates at 45 °C with a three-fold increase during rapid heating, but with a ten-fold increase for gradual heating. The results indicate that these biomolecules are less sensitive to rapid temperature changes between 32 °C and 40 °C. These findings accord with observations reported by Oehler-Janne et al. They highlighted that the pAkt(S473) is especially temperature-sensitive, whereas pAkt(T308) is stable [35]. After expanding the temperature range, we discovered that rapid heating to 45 °C increases pAkt(T308) phosphorylation.

A comparison of cell lines shows that most biomolecules behave similarly across tissues (liver and muscle). Despite that finding, the magnitude of the phosphorylation changes upon different stimulations. For example, IRS1 phosphorylation in the heating condition is observed more in C2C12 cells (2–4-fold change with different heating rates) than in the other two cell lines (maximally two-fold change). Unlike other cell lines, ERK1/2 in C2C12 cells is less temperature-sensitive throughout various testing conditions. Ultimately, the C2C12 cell line is of different tissue (muscle), whereas the other two cell lines are liver-originated. Additional studies must be conducted to investigate the underlying reasons for these different temperature sensitivities.

One major signaling pathway that is activated after insulin stimulation is the IRS1-PI3K-Akt pathway. The key biomolecules that are commonly used as indicators to evaluate this activation were examined in this experiment: IRS1, Akt, GSK3β, p70S6K, and FoxO1. The findings indicate a temperature-related increase in phosphorylation of IRS1, pAkt(S473), and GSK3β. From the immunofluorescence image, the Akt distribution in the rapid heating condition was observed to differ from the insulin activation. Akt is known to be recruited to the plasma membrane upon activation via the PI3K pathway [36,37]. Similar to the report, our observation of the insulin stimulation conditions reflects this Akt activation and translocation to the plasma membrane. On the other hand, in the rapid heating condition, the aggregated-like structure reflected the responses of Akt upon rapid heating to 45 ºC but lacked clear membrane recruitment. Previous studies have shown that PI3K is activated in a heat shock and is essential for Hsp90 downregulation and Hsp70 expression after cell stress [38,39,40]. Therefore, the observed aggregation potentially implies the activation of the PI3K-pathway-related recruitment of Akt. Additionally, Akt is also activated by cellular stress via a p53-dependent mechanism, which demonstrates a distinct spatial activation pattern compared to canonical activation [41]. Furthermore, it has been shown that Akt is activated by temperature stress to maintain metabolic functions in the liver [42], raising the possibility that a metabolic function difference among hepatocyte (Fao and HepG2) and myocyte (C2C12) reflects an Akt activation pattern difference. Further investigations are needed to confirm this Akt aggregation mechanism during rapid heating.

Contrary to the general trend of the increase in phosphorylation with temperature, p70S6K is dephosphorylated under rapid heating conditions as well as during gradual heating at 45 °C within 5 min [43]. Our results revealed that p70S6K is only phosphorylated from 10 min onward, with a gradual heating condition that is consistent with existing studies [44]. Because Akt activation was confirmed in both rapid and gradual heating conditions, the lack of phosphorylation of Akt downstream kinase, p70S6K, implies that p70S6K phosphorylation is also regulated by an Akt-independent mechanism. Studies have shown that heat-shock response pathway (HSP) activates the PI3K-Akt signaling pathway and results in Akt phosphorylation [29,45,46]. Western blot detection of Hsp70 also indicated activation of the heat-shock response pathway, which correlated with the trend of the phosphorylation increase of insulin biomarkers in rapid heating conditions. Aside from HSP-mediated pathway activation, p70S6K is also regulated in other mechanisms [38,44,47]. Therefore, the transient decrease in p70S6K phosphorylation is independent of HSP-mediated activation of Akt and potentially related to the rapid increase in phosphatase activity, such as PP2A [48]. Further investigation will be necessary to explore this transient p70S6K dephosphorylation behavior. Meanwhile, FoxO1 does not generally exhibit a significant phosphorylation change, which might also be regulated in other pathways. Additionally, an earlier time-course study showed that both Akt and p70S6K phosphorylation peaked at 15 min [38]. We demonstrated a pAkt(S473) phosphorylation peak and plateau from 20 min, whereas p70S6K phosphorylation increased continuously until 30 min.

In gradual heating conditions, most biomolecules are phosphorylated over time. The peak phosphorylation for IRS1, GSK3β, p70S6K, and FoxO1 was beyond the investigated 30 min duration. Future experiments with a longer duration will be able to elucidate the peak phosphorylation time points. In the gradual cooling experiment, some biomolecules displayed a transient increase in phosphorylation (IRS1 in Fao cells, GSK3β, and FoxO1 in all three cell lines) at the initial stage within 5 min, although this transient increase was not statistically significant. Thereafter, most biomolecules experienced a gradual increase in phosphorylation from 30 min with prolonged exposure to 25 °C where cell apoptosis accompanied by Akt activation would be initiated.

In short, this study has identified temperature-sensitive biomolecules in the insulin signaling pathways and their functioning thresholds. The variation in temperature change rates is expected to result in different activity statuses, among which p70S6K dephosphorylation observed in heating is markedly time-dependent. Moreover, pAkt(T308) is less temperature-sensitive than pAkt(S473). The results reveal that future studies should position the cells within 32 °C to 40 °C. Beyond that range, the effects of biomolecule activities should be considered from a technical perspective. Therefore, we would like to raise awareness of the temperature sensitivity of biomolecules and urge future NIR research designs to include temperature considerations.

## 5. Conclusions

The rapid growth in NIR optogenetic research in recent years highlights the necessity for deep-tissue in vivo optogenetic manipulation. Effects on signaling biomolecules exerted by rapid NIR-associated heating has not been studied adequately. We sought to investigate and to provide a pioneering insight into this area by examining the temperature-related phosphorylation of seven key biomolecules from the insulin signaling pathway in three selected cell lines originating from different mammalian species. Western blot results were quantified and analyzed statistically. A temperature sensitivity profile between 25 °C and 45 °C was built to show effects of the respective exposures to rapid and gradual temperature changes. The findings underscore the necessity for temperature monitoring in NIR-related research, especially when studying the signaling pathways that use phosphorylation as a key indicator for activity confirmation. These findings also expand our understanding of the thermal sensitivity of essential biomolecules in the insulin signaling pathway. Signaling pathway activation is a dynamic process for which multiple factors must be considered. Other pathways such as HSP are activated concurrently. Their respective effects require additional detailed analyses. Aiming at raising awareness of temperature-related issues, this work has also shed light to guide additional research to assess NIR thermal effects in other signaling pathways and to explore cross-effects of different signaling pathways in different cell lines. The information obtained by this study is expected to support the advancement of NIR optogenetic research.

## Data Availability

The data are available upon request from the corresponding author.

## Supplementary Materials

The following supporting information can be downloaded at: https://www.mdpi.com/article/10.3390/cells11193136/s1, Figure S1: Rapid temperature increase and its corresponding Hsp70 phosphorylation change.
