# Supplementary figures and images for: Systematic Interrogation of the Temperature Perturbation in the Insulin Signaling Pathway for Optogenetic Stimulation
Source: Cells. 2022 Oct 5;11(19):3136. doi: 10.3390/cells11193136 (PMC9564263; doi:10.3390/cells11193136)

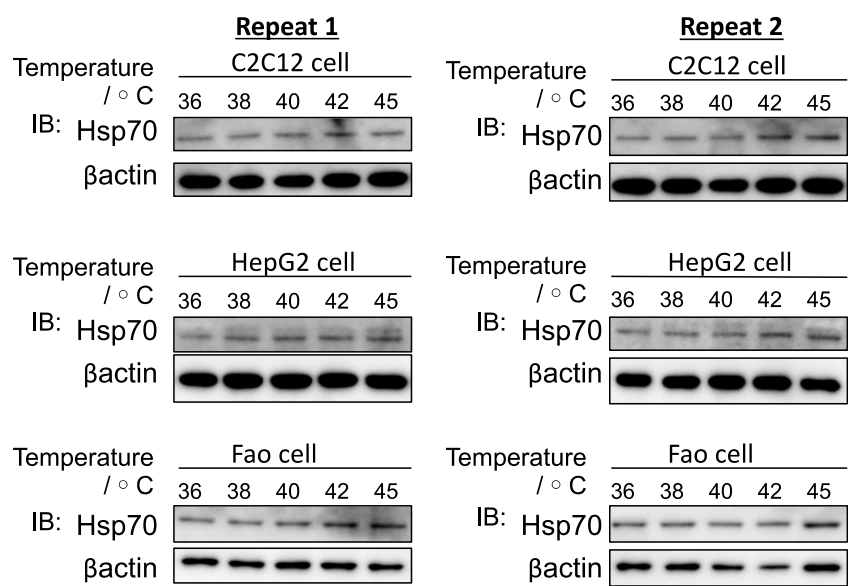

**Figure S1.** Rapid temperature increase and its corresponding Hsp70 phosphorylation change.

Supplement: Supplementary file 1 [file cells-11-03136-s001.zip › cells-1898554-supplementary.pdf]
